# Arthroscopic Single-Bundle Anterior Cruciate Ligament Reconstruction Using the Quadrupled Hamstring Tendon Graft: A Single-Institution Experience From North-Eastern India

**DOI:** 10.7759/cureus.40547

**Published:** 2023-06-17

**Authors:** Naren P Khatri, Iran Bharali, Imran Khan, Gauri S Borgohain

**Affiliations:** 1 Orthopaedics, Down Town Hospital, Guwahati, IND; 2 Orthopaedics, Assam Medical College, Dibrugarh, IND

**Keywords:** anterior cruciate ligament (acl), north-east india, north-eastern india, arthroscopy surgeon, semitendinosus graft, single bundle anterior cruciate ligament reconstruction, anterior cruciate ligament reconstruction (aclr)

## Abstract

Background

There is a lack of literature regarding the arthroscopic approach to a single-bundle anterior cruciate ligament (ACL) reconstruction using the quadrupled hamstring tendon graft in the north-eastern Indian population.

Methodology

A prospective, single-center study was planned for patients with ACL tears according to the eligibility criteria and with a defined surgical protocol. Patients were followed up from the preoperative period for at least one year, and knee function was evaluated using the International Knee Documentation Committee (IKDC) subjective knee score and the Lysholm knee score.

Results

A total of 29 patients were followed up for a mean of 14.6 months (12-22 months). The mean age of patients was 26.83 ± 7.50 years, with a male:female ratio of 4.8:1 and almost equal involvement of both knees. There was statistically significant improvement (p<0.001) in results in the Lachman test, anterior drawer test, pivot shift test, IKDC score, and Lysholm score. No intraoperative or postoperative complications were found in the present study.

Discussion

The study shows that arthroscopic anatomical single-bundle ACL reconstruction using quadrupled hamstring tendon grafts is a minimally invasive, safe, and effective procedure that provides anteroposterior and rotational stability and good to excellent functional outcomes.

## Introduction

The anterior cruciate ligament (ACL) is the primary and predominant stabilizing structure of the knee that resists the anterior translation of the tibia over the femur. ACL injuries generally occur in sports activities, motor vehicle accidents, and falls, with the reported incidence of ACL injuries being 2.8% of the healthy athlete population [1].

The overall annual incidence of ACL tears was found to be 68.6 per 100,000 people [2]. In a north Indian epidemiological study [3], ACL injuries were the most common sports injuries with 86.5% (n=314), followed by meniscal injuries in 78.24% of participants. Non-contact mechanisms led to ACL injuries in 70% of cases, while the remaining 30% were from direct contact [4,5]. Usually, 5% of acute ACL injuries are often accompanied by lateral meniscus injuries, with the medial meniscus at 45%, followed by injury to the medial collateral ligament at 34% [6,7]. These injuries result in acute and chronic pain, joint effusion, muscle weakness, altered movement, and reduced functional performance, and can lead to long-term undesirable clinical sequelae like meniscal tears and chondral lesions, eventually leading to early-onset posttraumatic osteoarthritis (OA) [8,9].

Treatment consists of conservative management or surgical intervention, with the latter being considered by young patients who are resistant to modifying their energetic lifestyle and have a keen interest in heavy work, sports, or recreational activity. ACL repair was first described in the early 1900s, and initially, it was performed by reapproximating the ruptured ACL ends with sutures [10-13]. The high chances of failure (40%-100% failure of ACL healing even after surgical approximation with sutures) led to the abolishment of the technique [9,11,14-16].

The evolution of ACL reconstruction (ACLR) has enabled a return to pre-injury activity levels. ACL reconstruction (ACLR) is characterized by debridement of the torn ends of the native ACL, followed by reconstruction of the new ligament by using common grafts such as plain hamstring tendon, bone-patella tendon-bone graft, quadrupled hamstring tendon graft, quadriceps graft, or synthetic [17]. Patients with patellar tendon grafts usually have more anterior knee pain due to the trauma to the extensor mechanism, and hamstring tendons have low donor morbidity [18]. Drilling of the tunnel is also a topic of debate, as growing literature has expressed a few concerns about the difficulty in achieving physiological and anatomical reconstruction with transtibial drilling of femoral tunnels [19].

The anatomic ACL reconstruction concept attempts are aimed at closely reproducing the patient's anatomy. Anatomic ACL graft placement is defined as positioning the ACL femoral and tibial bone tunnels at the center of the native ACL femoral and tibial attachment sites [20]. In this technique [20], the femoral tunnel is at a lower position, e.g., the left knee at two o’clock, offering more rotational stability than a higher position, e.g., the one o’clock position. There has been debate in the literature regarding the superior knee stability in the double-bundle technique; however, some studies showed little or no significant difference between anatomical single-bundle or double-bundle techniques [21-27].

The paucity of data in the north-eastern region of India regarding the outcome of anatomical single-bundle ACL reconstruction using quadrupled hamstring graft (HTG) encouraged us to plan this prospective observational study, aiming at the evaluation of the functional outcome and stability of the knee following arthroscopic anatomical single-bundle anterior cruciate ligament reconstruction using quadrupled HTG.

## Materials and methods

This study was designed as a single-center, prospective, single-arm cohort study performed following the revised Declaration of Helsinki guidelines regarding medical research. This trial was carried out strictly according to the Strengthening the Reporting of Observational Studies in Epidemiology (STROBE) guidelines [28] after obtaining ethical clearance from the Institutional Ethical Committee (Down Town Hospital IRB 0031/16).

Study participants and settings

All the patients with ACL tears presenting to the emergency or outpatient department of orthopedics at Down Town Hospital, Guwahati, Assam, India, were analyzed, and the patients were included according to the eligibility criteria.

Eligibility criteria

Young patients with American Society of Anesthesiology (ASA) I and II scores (active with a future interest in professional or recreational sports or who are involved in vigorous activities but are unwilling to change their active lifestyle), patients with ACL injury (usually after four weeks of injury when the acute inflammatory phase of the injury has subsided and a full range of motion and good quadriceps strength have been regained with no extensor lag), skeletally mature with a closed epiphyseal plate, with no history of prior knee surgery, and clinical, radiological, and arthroscopic evidence of ACL deficiency were included in the study. Written informed consent was taken from each participant before the commencement of the study.

Patients with ACL injuries with associated intra-articular fractures, osteoarthritic changes in x-ray, an anterior cruciate ligament tear associated with other ligament injuries in the same knee, and patients with bilateral ACL tears were excluded from the study.

Sample size calculation

A maximum permissible convenience sample within a stipulated time frame was included in the study.

Intervention

**Figure 1:**
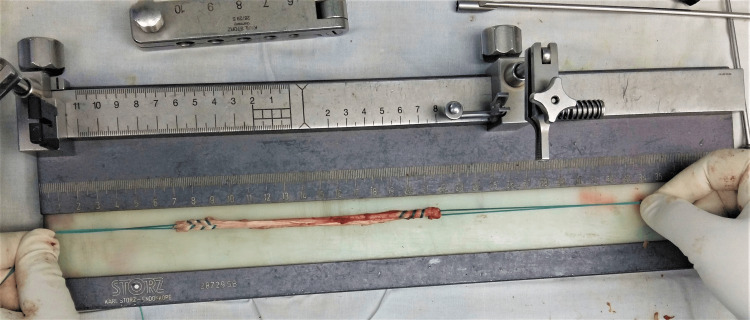
Prepared semitendinosus graft

All surgeries were performed by a single senior surgeon using a 4 mm and 30-degree arthroscope from Anthrex. After diagnostic arthroscopy, the ipsilateral semitendinosus tendon and gracilis (if the length of the semitendinosus is less than 260mm) tendon were harvested. A Krackow-type whipstitch on both ends of the tendon was placed, and the tendon was further folded to make four strands. The femoral tunnel is drilled through the independent anteromedial portal. The beath pin was placed through the femoral aimer, aimed at the center between the anteromedial (AM) and posterolateral (PL) bundle attachments at two o'clock on the left side and 10 o'clock on the right side. The femoral tunnel is reamed over the beath pin with a 4.5-mm reamer. A socket was reamed according to graft length and diameter. The tibial tunnel was made using the tibial guide at an angle of 50 to 55. The guide was placed posteriorly over the stump footprint. The guide pin was drilled and brought through the stump. The tibial tunnel was reamed according to the diameter of the graft. The intra-articular edges of the tunnels were smoothed using the automated shaver. The femoral end of the graft was marked for flipping the endo button using the surgical marker. The EndoButton was passed through Ethibond. The Ethibond was gradually pulled from the lateral aspect of the thigh, and the graft had been visualized by the camera. The EndoButton was flipped and fixed at the lateral femoral cortex. After cycling the loading of the knee for three minutes, a bio-absorbable screw 1 mm smaller than the tunnel was used for tibial fixation in 30-degree flexion. Figure 1 shows the prepared semitendinosus graft.

Physiotherapy was started on the first postoperative day according to the ACL rehabilitation protocol. All the patients were followed up on the seventh day for dressing. Staple removal was done on the fourteenth day. The periodic evaluation was done at six weeks, 12 weeks, six months, and one year, respectively.

Outcome assessed

All baseline demographic variables, including age, gender, mode of injury, knee involvement, mean time of operation from injury, and associated meniscus tear, were noted preoperatively. Knee function was evaluated preoperatively, at six months, and one year after operation using the International Knee Documentation Committee (IKDC) subjective knee score and Lysholm knee score. The final score at the endpoint of the study (one year) was compared with the preoperative score.

Statistical analysis

The collected data were tabulated in an Excel sheet, and statistical analysis was performed in Statistical Package for Social Sciences (SPSS) version 22 for Windows; SPSS Inc., Chicago, USA. The data were expressed as mean ± standard deviation. Categorial data were compared using the Chi-square test, while the difference between means was evaluated using the Student’s t-test, and the level of significance was taken at p ˂0.05.

## Results

**Figure 2:**
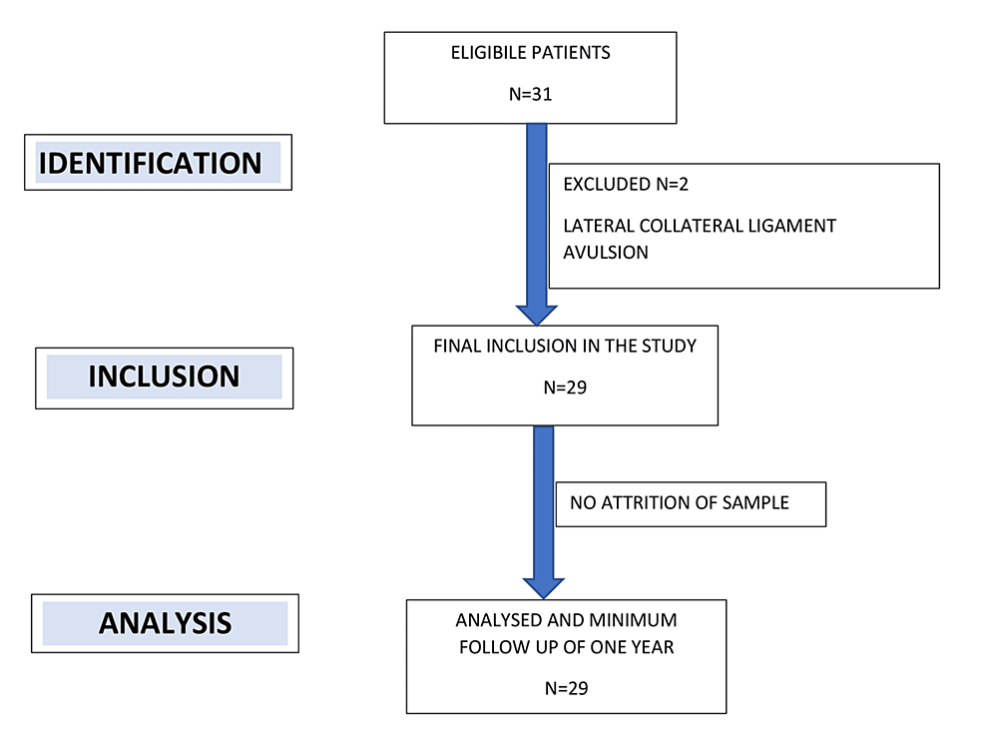
Flowchart of the study

The overall study design is shown in Figure 2.

A total of 31 patients who underwent ACL reconstruction during the scheduled period were analyzed for the study. Out of those two patients, one had a lateral collateral ligament avulsion. Excluding those two patients, 29 (24 males and five females) patients were included in this study. Demographic data and baseline demographic details are enumerated in Table 1.

**Table 1:** Demographic parameters of the patients

Demographic parameters		Results
Age (in years)	Mean± standard deviation	26.83±7.50 years
	Range	16-44 years
Gender	Males	24 (82.76%)
	Females	5 (17.24%)
Knee involvement	Right	14 (49.28%)
	Left	15 (51.72%)
Mode of injury	Sports	18 (62.07%)
	Domestic fall	7 (24.14%)
	Road traffic accident	4 (13.79%)
Associated tear	Lateral meniscus	9 (31.01%)
	Medial meniscus	8 (27.53%)
	Bilateral meniscus	1 (3.45%)
Time between operation and injury	Mean (range)	5.24 months (1-24 months)
Graft harvested	Semitendinosus	28 (96.55%)
	Both gracilis and semitendinosus	1 (3.45%)

Preoperatively, the Lachman test was grade II positive in four (13.79%) patients and grade III positive in 25 (86.21%) patients. At the end of the study, the test was negative in 28 (96.55%) patients and grade I positive in one (3.45%) patient, which is statistically significant (p-value<0.001) 

Preoperatively, the anterior drawer test was grade II positive in eight (27.59%) patients and grade III positive in 21 (72.41%) patients. At the end of the study, the test was negative in 28 (96.55%) patients and grade I positive in one (3.45%) patient, which is statistically significant (p-value<0.001)

**Figure 3:**
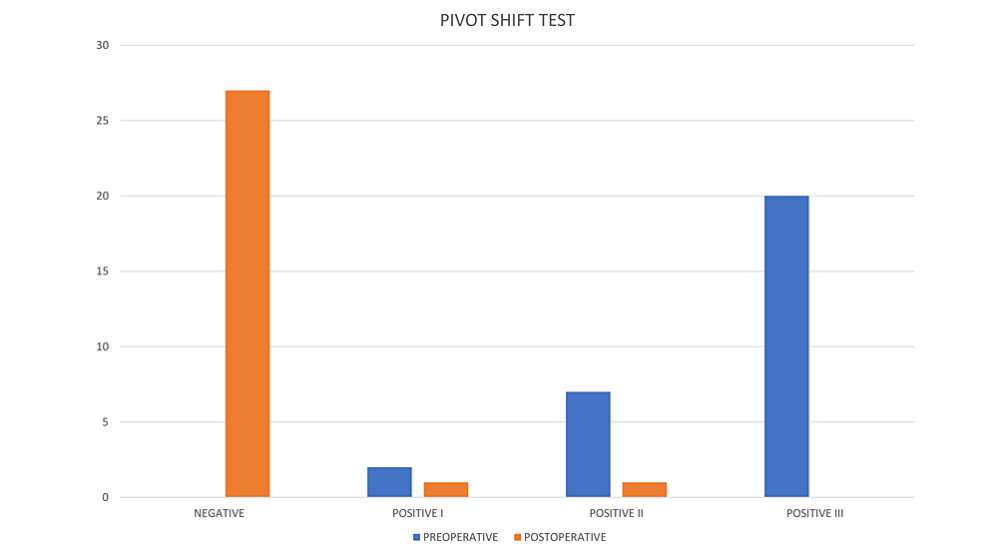
Bar diagram depicting pivot shift test at preoperative period and at one-year follow-up

Preoperatively, the pivot shift test, as shown in Figure 3, was grade I positive in two (6.9%) patients, grade II positive in seven (24.14%) patients, and grade III positive in 20 (68.96%) patients.

At the end of the study, the test was negative in 27 (93.10%) patients, grade I positive in one (3.45%) patient, and grade II positive in one (3.45%) patient, which is statistically significant (p-value<0.001).

**Figure 4:**
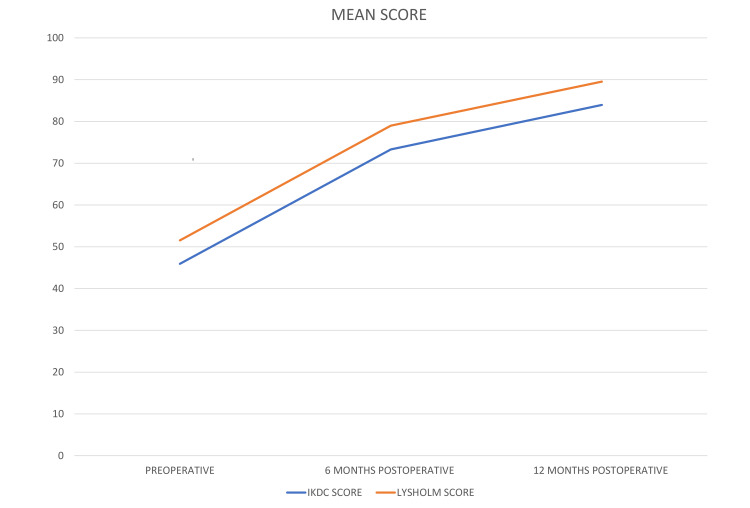
A line diagram depicting the IKDC score and Lysholm score at different time intervals

There was a significant improvement in IKDC score postoperatively (p-value <0.001) as shown in Figure 4, from 45.93 ± 10.97 preoperatively to 73.30 ± 8.47 (at six months) and finally 83.94 ± 7.95 (at 12 months).

Similar results were found in the Lysholm score, as shown in Figure 3, showing significant improvement in the Lysholm score postoperatively compared to preoperatively (p-value <0.001). There were no intraoperative or postoperative complications.

## Discussion

The ACL provides the primary resistance to anterior translation of the tibia as well as internal rotation of the tibia. There are a plethora of management options available, ranging from conservative management to surgical intervention, depending on the patient’s choice and the amount of instability. In our study, we managed ACL tears by anatomical (using independent transportal femoral tunnel) single-bundle ACL reconstruction using quadrupled hamstring tendon autograft (HTG) with femoral fixation with EndoButton and tibial fixation with bio screw.

The baseline demographic data, i.e., mean age, ranged between 26.83 ± 7.50 years, which was similar to previous studies [29-31]. The age group of fewer than 30 years comprised 22 patients (75.87%), confirming the association of ACL years, especially in young individuals. There was more male predominance in the study as men are mostly involved in sports activities and motor vehicle accidents than females, especially in a developing country like India, which is similar to other Indian studies [19,20]. But as we compare with the Western world, the ratio of males to females decreases, which can be explained by the increased involvement of females in sports and biking.

There was almost equal involvement of the right and left knees in the present study. However, there is no predilection for site involvement in the literature [32]. In our study, 62.07% of patients sustained an injury during sports, while road traffic accidents (RTA) constituted 13.79% of the mechanisms of injury. The rest of the patients (24.14%) could recall a history of falls from stairs or on level ground that incited their symptoms. This is similar to the studies of other authors like Patond et al. [33] and Jones KG [34]. However, RTA was the most common mode of injury in the studies of Zhang et al. and Kumar et al. [29,31]. Another observational study by Sayampanathan et al. concluded that 82.4% and 17.6% of ACL tears were sports- and non-sports-related, respectively [35]. Soccer, basketball, volleyball, and racquet games were the top four sporting activities causing ACL tears. The mean time between injury and treatment in the present study was 5.24 months, which was similar to other studies [29,31]

ACL injuries may also be associated with meniscal tears or meniscal injuries that can present over a period of time due to faulty weight distribution due to ACL tears. This has been supported by the fact that in untreated ACL-deficient knees, the incidence of meniscal lesions increases with time [36]. In our study, a concomitant meniscal tear was found in 62.07% of patients, with the lateral meniscus being more commonly involved than the medial meniscus. These results were in contrast to Lewis PB et al. [37], who reviewed 11 articles on single-bundle ACL reconstruction that showed 39% medial meniscal tears, 34% lateral meniscal tears, and 2% both meniscal tears. However, this finding of medial versus lateral meniscus involvement may be incidental, or it is difficult to comment with such a small sample size.

Many autografts and allografts, mainly including HTG, bone-patellar tendon-bone (BPTB), quadriceps tendon with or without bone graft, tibialis anterior and posterior, Achilles tendon, etc., have been used in the literature [38]. The ideal graft to be used for ACLR should exhibit similar properties to the native ligament, minimize donor site morbidity, and allow secure fixation as well as early incorporation [39]. The BPTB autograft has been considered a benchmark graft in the literature, consisting of the central portion of the patellar tendon along with its bone plugs from the tibia as well as the patella [38]. However, it can lead to anterior knee pain [40,41]. To overcome the disadvantages of BPTB grafts, HTG came as a better option, as these can be folded onto themselves to form two to six strands, increasing the graft diameter and strength. Another option for ACLR is using a versatile quadriceps tendon graft; however, that too can lead to certain complications like intraoperative bleeding because of violation of the quadriceps muscle, retraction of the rectus femoris, and chances of fractures of the patella [42].

Vinagre et al. explained different HTG preparation techniques for ACLR and concluded that preparation techniques depend on patient characteristics and surgeon preference [43]. In recent clinical practices, transportal (TP), as well as all-inside techniques (AIT), are the most common techniques used, which have shown no significant difference in postoperative functional outcomes [44]. The anteromedial portal in TP advocates the optimal setting of ACLR, leading to improved stability; however, AIT is a newer technique that is still under evaluation for the bungee cord phenomenon [44].

In our study, there were no intraoperative and postoperative complications similar to those reported by Zhang et al. [29] and Mathai et al. [30]. Kumar et al. [31] reported complications in seven patients (stiffness in two patients, infection in two, femoral tunnel blow-out in one, and graft re-rupture in one patient) with a minimum of 24 months of follow-up, which is longer than our study. Lewis PB et al. [37] reported only three (0.29%) operative complications in 1024 patients: a blow-out fracture of the posterior femoral tunnel, a saphenous nerve injury, and a sartorius tendon rupture during graft harvesting. The same study reported a postoperative complication (debridement, infection, and manipulations) rate of 6% (52/921) in single bundle reconstructions and a graft failure rate of 4% (32/921).

The Lachmann test is the most accurate test for detecting ACL injuries, followed by the anterior drawer test and the pivot shift test [37]. We have used the entire trio of diagnostic tests for confirmation of ACL injuries in our patients. Also, the IKDC score and Lysholm score were used for comparison of the results, as these scores have been widely accepted and are proven to be better rating systems. Anteroposterior stability was improved similarly in all studies evaluated by Lachman and the anterior drawer test. The Lachman test was negative in 28 (96.55%) patients in our study, 31 (93.94%) patients in Zhang et al. [29], and 55 (88.70%) patients in Kumar et al. [31], postoperatively. Rotational stability had been significantly improved as evaluated by the pivot-shift test, which was negative in 27 (93.01%) patients in our study, 31 (93.94%) patients in Zhang et al. [29], and 59 (95.17%) patients in Kumar et al. [31], postoperatively. Based on the IKDC subjective score and Lysholm score, there is a significant improvement in postoperative scores compared to preoperative scores in all the studies.

There are a few surveys regarding the preference of Indian surgeons for ACL reconstruction. In 2008, Sandhu JS et al. [45] surveyed operative techniques and protocols, and it was found that 50% of surgeons preferred to wait for three to six weeks before performing surgery, and the single-incision arthroscopic technique (62.5%) was frequently used, followed by the two-incision arthroscopic technique (29.2%). The main choice of the graft was the semitendinosus or gracilis tendon, while the two main fixation methods used were bioabsorbable screws and interference screws [45]. The survey concluded that general consent was seen among the Indian surgeons concerning the surgical treatment and postoperative protocols, but vast variations concerning the selection of the graft and its fixation methods were present [45]. Another survey in 2015 by Vaishya R et al. [46] found that hamstring tendon grafts were preferred by 83.3% of surgeons, with 83.33% preferring single-bundle reconstruction. The most preferred fixation devices were interference screws on the tibial side and EndoButtons on the femoral side, which are preferred by 95.83% and 93.75% of surgeons, respectively. A total of 97.9% of surgeons use the outside technique for tibial drilling, and 89.6% of surgeons prefer an anteromedial transportal approach for femoral tunnel preparation. The latest survey in 2023 [47] concluded that the most common graft choice was the hamstring tendon (94%), and suspensory fixation on the femur side and interference screw on the tibial side (80%) were the most common fixation methods.

Our study has a few strengths, as this is one of the few studies that has generated data about surgical practice in north-eastern India. The surgical techniques that have been used are well-established in the literature, and we completely adhered to the study protocol. Also, we were able to interpret our results by comparing them with other studies published in the literature using similar methods.

However, the limitations of our study were the relatively small sample size done at a single center, the short follow-up time, and the lack of comparison groups. Secondly, long-term follow-up data are absent, because of which we cannot comment on the development of degenerative changes in the knee joint of operated patients.

## Conclusions

The study shows that arthroscopic anatomical single-bundle ACL reconstruction using quadrupled HTG is a minimally invasive, safe, and effective procedure that provides anteroposterior and rotational stability and excellent to good functional outcomes. The results in north-eastern India are similar to the national standards, and we can conduct future studies with elaborative sample sizes and comparative groups.
